# The New York State Board of Medical Examiners

**Published:** 1887-08

**Authors:** 


### *THE NEW YORK STATE BOARD OF MEDICAL EXAMINERS.*

SIR: A law of 1874 established for the State of New York a State Board of Medical Examiners. Frequent deaths and occasional resignations changed its original membership several times, until, upon the recommendation of the undersigned (who, therefore, objected repeatedly to his own appointment) and for reasons easily understood, all the members of the present Board, with one exception, were selected by the Honorable the Board of Regents of the University of the State of New York from amongst the medical men of Albany, the seat of the government and the Board of Regents.

The profession never expected the law as it was passed in 1874 to be efficient. It was believed by many that some of the medical colleges objected to the establishment of a State Board altogether, though others were known to favor it. It was certain that sectarian influences succeeded in undermining the passage of the original bill and emasculating it. *It is certain* that no State Board of Examiners will ever benefit either the profession or the public, both of which stand in equal need of it, before the license to practice medicine will depend on the *compulsory* passing of a successful examination before the State Board. As the law stood, nobody ever applied for examination and the degree of M. D. of the University of the State of New York, who was in the possession of a diploma from a college in good standing. Such few as volunteered to come forward, were men who had previously failed in their college examination, or "practised medicine" without study, knowledge, or any title whatsoever. There being no rules and regulations referring to a minimum of accomplishments or requirements, a few of these were let loose upon the unsuspecting public with a diploma; the majority, however, failed.

When the new Board was appointed in the beginning of this year, its members accepted their positions upon the condition that the Board of Regents would authorize a number of rules and principles which were to regulate the examinations and the granting of degrees. As they have been approved by the Regents, I am directed by the Board of Examiners to present them to you for your information, and, if you deem proper, for publication and comment. We know quite well that, as long as the examination by the State Board is not made *compulsory*, any number of rules and principles will prove their inadequacy and inefficacy again and again. But the present Board hopes that its earnest recognition of the rights and dignity of medical science, art and practice will be admitted by, and found acceptable to the profession, and that the latter, after a minimum of requirements for the admission into the ranks of the profession has been officially accepted by the Regents, will feel encouraged to continue its exertions.

in behalf of both the elevation of the standard of medical education and the protection of the public.

Not one of the recent applicants for a degree has proved successful. One of them had failed in his college examination a few weeks previously, and now threatens to swell the number of graduates of the "University" of a neighboring State.

Very respectfully,

A. JACOBI, M. D.

110 WEST 34th ST., NEW YORK, July 25, 1887.

*Rules for Examination.*

The members of the State Board of Medical Examiners accept their positions with this understanding :

A candidate for the degree of Doctor of Medicine to be given by the Board of Regents, either desires an additional degree after he has received his diploma from a chartered medical college, or he has no diploma from any chartered medical college, and desires or prefers one from the Board of Regents. The degree given by the Board of Regents is to be, or become, an honorable distinction. It must be the object of the law to protect the people and to ennoble the medical profession, and not to facilitate the entrance into it of persons unfit or unqualified. The profession does not require larger numbers, but does insist upon an elevated standard. Therefore, the examination must be strict, and must be conducted according to the following rules :

1st. The examinations before this Board shall be conducted in the English language exclusively.

2d. The candidate shall be allowed two and a-half hours for each examination. The examination shall be in writing. The candidate must not consult books, extracts, notes, or persons, and must not communicate with any one during the two and a-half hours allotted to him. To do so is to be considered a failure to pass.

3d The examination in clinical medicine and in clinical surgery shall consist in the actual examination of patients by the candidate, and a discussion in regard to the diagnosis, prognosis, and treatment of the cases.

4th. The examination in chemistry shall include the actual testing of a specimen of urine, in regard to its reaction, specific gravity, and the presence or absence of albumen and sugar.

5th. Each Examiner shall have the privilege, if he so desire, of supplementing his written examination by an oral one, in the presence of two other members of the Examining Board.

6th. The scale of marks shall be from zero to ten; ten being perfection, and anything below six being a failure to pass the examination.

7th. The questions and answers with their marks shall remain in the possession of the Board of Regents, and shall be open to inspection.

8th. When the candidate shall have completed all his examinations, the Board of Examiners shall meet and hear the result of the examination in each branch. And within ten days thereafter, each member of the Board shall make a written report as to the merits and acquirements of the candidate; being guided in this report, not alone by the result of the examination in his particular branch, but also by the result of the examinations in the other branches. And each member of the Board shall send his report, together with the questions and their answers and their marks in his branch, to the Secretary of the Board of Examiners, to be by him transmitted to the Secretary of the Board of Regents.

And, furthermore, it is the opinion of the Board of Examiners, that in order to receive the degree of Doctor of Medicine, the candidate should successfully pass in every branch, or at least in every branch but one.

*State Board of Medical Examiners.*

*President*—Abraham Jacobi, M. D., Examiner in Pathology.

*Vice-President*—Albert Vanderveer, M. D., Examiner in Surgery, and Clinical Surgery.

*Secretary*—Henry Hun, M. D., Examiner in Clinical Medicine, and in Materia Medica and Therapeutics.

James P. Boyd, M. D., Examiner in Obstetrics.

Franklin Townsend, M. D., Examiner in Physiology.

Samuel R. Morrow, M. D., Examiner in Anatomy.

William Hailes, Jr., M. D., Examiner in Histology.

Willis G. Tucker, M. D., Examiner in Chemistry.

[As will be seen, nearly all the above are professors of the Albany Medical College. They are men committed to the policy of higher medical education. Instead of licensing unqualified men they doubtless will send many back to the colleges for further study. Let us hope that such a board as this will be given power to compel all persons to come before them for examination before they can practice.—Ed.]

